# Exploring Attachment to the “Homeland” and Its Association with Heritage Culture Identification

**DOI:** 10.1371/journal.pone.0053872

**Published:** 2013-01-23

**Authors:** Nelli Ferenczi, Tara C. Marshall

**Affiliations:** 1 Department of Psychology, Brunel University, London, United Kingdom; Umeå University, Sweden

## Abstract

Conceptualisations of attachment to one's nation of origin reflecting a symbolic caregiver can be found cross-culturally in literature, art, and language. Despite its prevalence, the relationship with one's nation has not been investigated empirically in terms of an attachment theory framework. Two studies employed an attachment theory approach to investigate the construct validity of symbolic attachment to one's nation of origin, and its association with acculturation (operationalized as heritage and mainstream culture identification). Results for Study 1 indicated a three-factor structure of nation attachment; the factors were labelled secure-preoccupied, fearful, and dismissive nation attachment. Hierarchical linear modelling was employed to control for differing cultures across participants. Secure-preoccupied nation attachment was a significant predictor of increased heritage culture identification for participants residing in their country of birth, whilst dismissive nation attachment was a significant predictor of decreased heritage culture identification for international migrants. Secure-preoccupied nation attachment was also associated with higher levels of subjective-wellbeing. Study 2 further confirmed the validity of the nation attachment construct through confirmatory factor analysis; the three-factor model adequately fit the data. Similar to the results of Study 1, secure-preoccupied nation attachment was associated with increased levels of heritage culture identification and psychological well-being. Implications of the tripartite model of nation attachment for identity and well-being will be discussed.

## Introduction

*A person without a motherland is like a nightingale without its song.*

Russian proverb

### Introduction

Social construction of one's nation of origin as an object of primordial attachment renders emotional ties similar to that of kinship [1], and an individual's national identity becomes tied symbolically to family. It is a testimony of the acceptance of this concept in the prevalence of words such as ‘motherland,’ and ‘fatherland’ in numerous languages. In his speculation of the ontology of national identity, Smith [2] observed that the “family of the nation overrides and replaces the individual's family but evokes similarly strong loyalties and vivid attachments” (p. 79). If attachment to nation of origin was situated in terms equivalent to that of attachment theory in the consciousness of individuals, then a richer understanding of the construct could be elucidated. The attachment paradigm centres on the characteristics of the bond between an infant and her/his primary caregiver, which influences the former's behaviour and perceptions of the self and of others [3]. At present, there have been no investigations into whether attachment to one's nation of origin can be interpreted within this framework, despite linguistic and conceptual links. The goal of the present research was to bridge this gap in an effort to contribute to the understanding of the relationships that individuals form with their nation of origin. We first review attachment theory and its varying models, then analyse place attachment.

### Attachment Theory

Attachment theory investigates the bonds that infants develop with their primary caretaker [3]. Six criteria of defining attachment bonds across the life span were outlined by Ainsworth [4]: desire for maintaining proximity, distress at involuntary separation, holding emotional significance, providing security and a safe haven in times of distress, persistence, and specificity to a particular individual. The framework was further extended into the social psychology discipline by Hazan and Shaver [5] to examine adult romantic relationships; such cross-pollination has been fruitful enough that research on adult and adolescent close relationships relies heavily on attachment theory.

Several attachment models dominate the research literature. A two-dimensional model of attachment [6], [7], [8], wherein the latent models of self and other, derived from Bowlby's [9] theoretical discourse of internal working models that individuals develop through attachment in childhood, has yielded four categories of attachment: fearful (characterised by both negative self and other models), dismissive (characterised by a negative model of other and a positive self model), preoccupied (embodying a negative model of self and a positive other model), and secure (holding both positive self and other models. Notwithstanding the theoretical dispute regarding optimal measurements of attachment [10], [11], and the array of instruments to capture the varying dimensions of attachment [12], attachment orientations have been associated with numerous cognitive components [13], [14], [15], behavioural outcomes [16], [17], [18] and affective variables [5], [19].

The attachment framework can be applied to various attachment figures in an individual's life [20], [21], [22], [23], [24]. Investigation of attachment figures outside of the habitual figures of parent and romantic partners has often been neglected [25]. In a comprehensive review of attachment research, Shaver and Mikulincer [26] called for an expansion of investigation on the symbolic relations between political leaders and their followers. The aim of the present research was to investigate a conceptually similar symbolic attachment that individuals form to the nation of their origin. We drew on theories of place attachment to formulate the nation attachment framework.

### Attachment to Place

Place attachment can be defined as a social attachment between an individual and a place [27], and has been mostly investigated within the realm of environmental psychology. Place attachment has been measured in terms of behavioural, affective, and cognitive components [28]. It is linked with place identity, which has been conceptualised as a process of self-categorisation [29]. The distinction between place attachment and the current research lies in the former centring most commonly on neighbourhood attachment and neglecting attachment to a higher-order category [27], [30].

Dixon and Durrheim [31] called for cross-fertilization between environmental and social psychology as they postulated that the former's consensus that place and self mutually influence one another directly challenges the “disembodied notion of self” (p. 40) that social psychology propagates. Places are conceived as “dynamic arenas that are both socially constituted and constitutive of the social” (p. 27) [31]. The present studies build on this framework that nations are socially constituted entities that are comprised as symbolic attachment figures. This would imply that nation attachment would be strongest for those individuals who held defined nation concepts in their cognitive schemas, as language has been postulated to be the force that binds people to places [32]. Socially constructed symbols such as nations, particularly nations that individuals identify with, can then be conceived not only as social places, but as places of attachment.

### Attachment to Nation

An extensive literature review in major psychological databases revealed that no existing research to date has been conducted on interpreting the relationship that individuals form with their nation through an attachment theory perspective. Research has investigated the significance of identification with one's group [33], and extended it to identity fusion with one's group, wherein the boundaries between the personal and social selves can become blurred [34]. Smith and colleagues [35] investigated the attachment that one forms to social groups. Results implied the presence of two attachment dimensions, anxiety and avoidance, which were distinct and separate from other measures of relationship attachment and group identification, and predicted behavioural, cognitive, and affective components. The present investigation aimed to shift from the micro level of voluntary group membership that was the focus of that study to the macro level of nationality group, which also spans over longer time periods and to which an individual tends to be socialised into from a young age. Furthermore, it is postulated that the crux of the attachment relationship lies not in the general attachment to a group, but in the specific and symbolic representation of the nation as an attachment figure.

Feshbach [36] developed a scale to distinguish nationalism and patriotism, which yielded five factors. Subsequently, attachment mechanisms in early childhood were compared to these indices of national attachment, yet the study confined itself to analysing bivariate correlations. The exact nature of the relationship, specifically the predictive power of parent attachment on national attachment could have been elucidated through the construction of a hierarchical regression model, but was neglected. Despite the inclusion of national attachment, a comprehensive literature review that aimed to underscore the fundamental similarities between the attachment that individuals form with their parents and their nation, and ample examples of parental imagery employed in nation conceptualisation, the study failed to interpret the results in terms of an attachment framework. Nation attachment, as a distinct attachment orientation separate from other relational bonds, remained absent in this study.

A keystone of the present research and nation attachment is the personification of nations as attachment figures, parallel to family, and specifically, to parents. Nations have been anthropomorphised in many disciplines and cultures, some of which will be further outlined below in terms of the six attachment bond criteria. Pivotal to the present proposition of symbolic nation personification, spontaneous anthropomorphisation of place has also been reported [37]. When participants were asked whether Canada, Quebec, and Canadian provinces were more like a body or a container, the authors reported that participants significantly conceptualised places as like a body, and that this relationship was mediated by nationalism, with more nationalistic individuals reporting higher levels of body imagery [37]. It was concluded that the symbolic significance of the body metaphor was unknown. The present research seeks to ameliorate this uncertainty through positing that the body metaphor stems from nations being conceptualised as attachment figures.

Attachment to nation, though neglected in empirical research, has often been documented in other disciplines. A brief review implies that integrating an attachment framework to investigate this construct would be fruitful. In terms of the six attachment criteria, specificity of the attachment is exemplified by the tendency of individuals to form an attachment to their specific nation of origin. Furthermore, nations have differing personifications, which has been observed in historical and sociological narrative, including the personification of India as a mother in the 19^th^ century [38], Africa as a mother when interpreting the status of women [39], and the Iranian ‘Vatan’ or homeland, conceptualised as a 600-year old mother [40], [41]. Nation personifications are persistent and are part of a long tradition in art, literature, sociological narrative, philosophical discourse, and folk traditions [36]. Persistence and proximity to the nation are also manifested in their grammatical engenderment in many languages, so that their personification is embedded in individuals' cognitive schemas. Nation personification has also notably influenced Spanish literature in its practice of romantic nation writing wherein Spain is personified as a mother and the citizens as her family [42]. It has also been reported that in Eastern European and Asia, national identity is constructed differently to the Western model, in that individuals experience a second birth into the social community and the native culture [2]. Thus, attachment to the nation is persistent and proximal from an individual's inclusion into the society, and is further reinforced by language.

The emotional significance of nation attachment has been frequently reported, particularly when interpreting individual motivations in war [1]. National attachment has also been investigated in terms of a symbolic affective attachment to the nation and its symbols and values [43], [44], implying that a degree of emotional significance is imparted to the bond that individuals form with their nation of origin. Nation imagery, such as that of the Vatan as a dying mother that has been failed by her children [41], and the symbolic fatherland which ethnically Hungarian groups, separated following the 1920 Treaty of Trianon from Hungary, construct [45], is created to elicit emotion [1]. Emotional significance of a nation and its perception as a secure base in times of distress can also be linked to its personification as a super family [1], [2]. It has also been observed that such personalization mechanisms account for why real families are put aside for the interests of an imagined one [1], which is postulated to stem from the perception of the nation as a secure base and thus can offer an insight as to why drastic measures are taken in its defence. Anxiety and separation, the final criterion of attachment, can be interpreted as the acculturative stress [46] that individuals experience when relocating outside of their nation of origin. Examples of separation anxiety are crystallised in the autobiographical narratives of Soviet exiles, who assembled a new consciousness of a son who “never comes back” (p. 511) [47]. Nation attachment can therefore be interpreted in terms of the six criteria of relationship attachment, and the present research aims to construct a model that will yield empirical support for the theoretical framework. In this vein, the nation attachment framework can also be employed to expound on the experiences of individuals residing in host cultures far from their symbolic attachment figure.

### Acculturation

Acculturation can be defined as a mutual process of cultural change resulting from continuous contact between two cultural groups [48], [49]. The maintenance of heritage culture and identity, and the adaptation to the new host (or mainstream) culture in the form of relationships sought among the larger societal group, are the two orthogonal factors that interact to produce measurements of acculturation [49], [50]. The resulting matrix denotes four distinct quadrants of acculturation strategy: separation (high heritage and low mainstream culture identification), assimilation (low heritage and high mainstream culture identification), integration (high levels of identification for both cultures), and marginalization (low levels for both).

Strong identification with both heritage and mainstream cultures (i.e. integration) has been widely conceptualised as the most positive acculturation strategy and marginalisation the least [49]. Research has consistently indicated that integration correlates most significantly with positive adaptation and psychological adjustment [48], conceptualised in terms of low depression scores and global mood disturbance [51], [52]. Subjective well-being (SWB) can thus be utilised as a proxy for psychological adjustment [53]. Conversely, acculturative stress, a symptom of low psychological adjustment, has been found to be associated with neuroticism [54]. Furthermore, weak identification with one's heritage and mainstream cultures (i.e. marginalisation) is correlated negatively with life satisfaction [55], SWB [56], mental health [57], self-esteem [58], sociocultural adaptation (defined as how well an acculturating individual adapts to daily life in a new cultural milieu), psychological adaptation [59], and continual displacement and loss in personal narratives [60].

General attachment tendencies have been linked to psychological and sociocultural adjustment in migrants [61], [62]. The affective, cognitive, and behavioural propensities that characterise differing attachment styles also shape the capacity for dealing with new cultural environments. Secure attachment has been found to be positively associated with integration, in particular with increased reports of contact and identification with one's host culture and heritage culture [61], and negatively with factors that are detrimental to psychological adjustment in a new culture [62]. The present research sought to extend these findings through investigating the specific symbolic attachment that individuals conceptualise with their nation of origin.

## Study 1

### Hypotheses

The first study endeavoured to illuminate whether individuals form symbolic attachments to their nations of origin, and whether such attachments can predict acculturation orientations that they adopt when adjusting to a new country and its culture. A cross-disciplinary venture would allow us to unpack the complex relationships and conceptualisations that individuals form regarding the nations they are from. Four hypotheses were generated.

#### Hypothesis 1

A framework of attachment to nation will emerge from the adapted relationship questionnaire that maps onto existing attachment models, paralleling the four factor model that Griffin and Bartholomew [7], [8] postulated of dismissive, secure, preoccupied, and fearful nation attachment.

#### Hypothesis 2

Nation attachment orientations that represent insecure attachment will be associated with general insecure attachment, confirming the construct's validity, and with increased neuroticism, replicating previous research findings of the positive association between general insecure attachment models and neuroticism [63]. Associations will corroborate the convergent validity of the derived nation attachment construct.

#### Hypothesis 3

Controlling for neuroticism and general romantic attachment, nation attachment orientations that endorse positive models of other will be associated with increased heritage and mainstream culture endorsement. Underlying negative models of other in attachment styles will be associated with decreased heritage and mainstream culture identification.

#### Hypothesis 4

Nation attachment orientations that are characterised by positive models of the self and/or other will be associated with SWB, after controlling for confounding variables.

## Method

### Participants

Factor analysis was conducted on 263 participants. Due to missing data, only 232 of the participants were retained for regression analysis (female: 126, male: 105, not stated: 1). Participants were between 16 and 65 years of age (*M* = 29.92, *SD* = 10.50). As the general experience of identification with heritage and mainstream cultures was of interest, participants were recruited from numerous countries. In terms of residence status, 35% (*N* = 82) of participants reported currently living in a country different to that of their birth for between three months and 41 years (*M* = 13.44, *SD* = 10.59), and 65% (*N* = 150) reported living in the country of their birth. Thirty-eight separate nations were represented, with the three most frequently cited nationality groups being European (37%), South Asian (22%), and North American (23%). Distribution of highest attained education was varied, with participants indicating that they had completed or were currently in their first degree (51%), had completed or commenced their postgraduate degree (33%), had completed secondary education (6%), or had completed a professional degree (10%). A high proportion of participants indicated that they were in full-time employment (61%); participants also reported being in full-time education (24%) or unemployed/retired (15%). Relationship status was also skewed with a significant amount of participants indicating that they were currently single (44%); participants reported other relationship statuses, such as being married (27%), in a relationship (16%), cohabiting (10%), or divorced/widowed (3%).

Socio-demographic variables were also compared in the two samples of those who reported residing in their country of birth, and those who resided in a host country. The two groups were homogenously distributed in terms of age, relationship, and employment status. There was a significant difference in education, with a higher proportion of participants who indicated they resided in their country of birth also indicating that they were currently undertaking their first Bachelor degree at university, χ^2^ (8, 230) = 17.48, *p* = .05, and gender, with a higher proportion of males citing residence in their country of birth, χ^2^ (1, 231) = 8.97, *p* = .05. Ethics approval for both studies was given by the Ethics Committee of the Department of Psychology at Brunel University, in accordance with the recommendations of the British Psychological Society. All participants provided informed consent prior to their participation in the online study, whilst retaining their anonymity to maintain confidentiality. Participants were given the opportunity to contact the researchers, refuse to participate, omit questions, or withdraw at any time without consequences.

### Materials

#### Nation Attachment

No existing instruments measuring attachment to nation of origin, conceptualised as similar to an attachment figure, were found. The Relationship Scales Questionnaire (RSQ) [7] is widely used in attachment research [11]. A 30-item scale that assesses Bartholomew and Horowitz's [6] four-category attachment prototype model, it has also been employed as a bi-dimensional measure of self and other models that individuals have internalised. The RSQ can be employed to assess three differing attachment constellations [5], [6], [64], along with four subscales that tap into their respective attachment styles: preoccupied, fearful, secure, and dismissive, as proposed by Griffin and Bartholomew [7].

The diversity of these items has resulted in a particularly versatile measure that can be assessed in accordance with each of the models from which it was adapted, along with a two-factor model of anxiety and avoidance [65]. Its plasticity designated it as an appropriate measure that could be adapted for the present research. As such, “country of origin” replaced any instances of “partner” to assess nation attachment. Participants were asked to indicate the extent to which each item was descriptive of themselves on a 5-point Likert scale (1 = *Not at all like me*, 5 = *Extremely like me*). Subscale reliability and sampled items are reported in the Results section.

#### Neuroticism

Neuroticism is positively correlated with insecure types of attachment [5], [63], and was thus included as a control variable and to assess the convergent validity of nation attachment. The neuroticism subscale from the Eysenck Personality Questionnaire – Brief Version (EPQ-BV) [66] was employed. Participants were posed 12 statements and asked to indicate the extent to which they were descriptive of themselves on a 5-point Likert scale (1 = *Not at all like me*, 5 = *Extremely like me*). A sample item asked “Do you worry too long after an embarrassing experience?” A robust alpha was obtained for this sample (α = .92).

#### Acculturation

Acculturation orientations were measured by means of the Vancouver Index of Acculturation (VIA) [50]. As acculturation is a bi-dimensional process wherein the relationship to the mainstream culture is distinct from the relationship to one's heritage culture [49], [50], [67] this instrument was deemed sufficient to capture both dimensions in the present sample in order to elucidate their association with nation attachment. Both subscales have 10 items each that tap into values, social relationships, and adherence to traditions, to which participants are asked to indicate their degree of agreement on a 9-point Likert scale (1 = *Strongly Disagree*, 9 = *Strongly Agree*). Sample items included “I often behave in ways that are typical of my heritage culture” and “I often participate in mainstream cultural traditions of my host culture” with ten statements mirrored so that they addressed both factors. Reliability alphas for this sample were robust for both heritage (α = .88) and mainstream culture dimensions (α = .89).

#### General attachment type

We included a measure of general attachment to romantic partners in order to establish the uniqueness of nation attachment, over and above that of this variable, when predicting variations in acculturation. Furthermore, significant correlations between general attachment type and nation attachment would serve to validate the latter scale. The Experiences in Close Relationships Scale – Short Version (ECR- S) [68] measures avoidant and anxious attachment styles. Both subscales have six items each, which refer to romantic partners in general, and are rated on a 5-point Likert scale (1 = *Strongly Disagree*, 5 = *Strongly Agree*). The anxiety subscale (α = .75) converges on perceptions of abandonment and need for reassurance; items include “I need a lot of reassurance that I am loved by my romantic partners.” Conversely, the avoidance subscale (α = .74) focuses on perceptions of desired distance in a romantic relationship, with a sample item reading “I try to avoid getting too close to my romantic partners.”

#### Subjective Well-Being

SWB was measured with the Satisfaction with Life Scale (SWLS) [69]. Five items (α = .86) assessed global life satisfaction, with statements such as “In most ways my life is close to my ideal” rated on a 5-point Likert scale (1 = *Strongly Disagree*, 5 = *Strongly Agree*).

### Procedure

Participants were invited to complete a study regarding attachment to nationality. The study was conducted online with the aid of Surveymonkey. The hyperlink to the survey was distributed via a London-based university intranet site, social networking sites, and psychology-oriented websites with an international range of participants. The link was also distributed through Amazon Mechanical Turk, with participants being offered $0.25 upon completion of the survey; IP addresses were inspected to insure no redundant data was present.

## Results

### Hypothesis 1: Nation Attachment

To test the hypothesis that a coherent construct of nation attachment could be derived, a principal component analysis (PCA) was conducted on the 30-item adapted RSQ questionnaire with oblique rotation (direct oblimin). Initial analysis revealed seven components with eigenvalues above Kaiser's criterion of 1. However, an inspection of the scree plot indicated that a three-factor solution would provide a best fit for the data, as inflexions justified the removal of components 4, 5, 6, and 7; retained factors are presented in Table 1. The components that were removed had eigenvalues below 1.25. A forced three-factor direct oblimin PCA was then conducted. Sampling adequacy was validated for analysis through the Kaiser-Meyer-Olkin measure, KMO = .91. Bartlett's test of sphericity indicated that correlations between items were sufficiently significant for PCA, χ^2^ (435) = 4287.56, *p*<.001. The three extracted components accounted for 53.58% of the variance in the model. Items retained had loadings above .55, with the exception of those items that were removed due to being cross-loaded onto two factors.

**Table 1:** Summary of Exploratory Factor Analysis Results of the Structure Matrix for the Country Attachment Questionnaire with Retained Factors (*N* = 263).

Items	Rotated Factor Loadings		
	Fearful	Secure-Preoccupied	Dismissive
I often worry that my country won't want to stay with me.	.82		
I worry about being abandoned by my country.	.80		
I often worry that my country doesn't love me.	.80		
I often worry about having my country not accept me.	.76		
I find that my country is reluctant to get as close as I would like.	.72		
I worry that my country doesn't value me as much as I value my country.	.68		
My country often wants me to be closer than I feel comfortable being.	.59		
I know my country will be there when I need it to be.		.77	
I want to merge completely with my country.		.75	
I am comfortable depending on my country.		.75	
I find it relatively easy to get close to my country.		.71	
I am comfortable having my country depend on me.		.69	
I prefer not to depend on my country.			.75
It is very important for me to feel independent from my country.			.71
I prefer not to have my country depend on me.			.65
I am comfortable without a close emotional relationship to my country.			.59
I find it difficult to depend on my country of origin.			.57
I am not sure I can depend on my country to be there when I need it to be.			.55
**Eigenvalues**	8.79	5.42	1.86
**α**	.88	.82	.79

The three factors were interpreted to reflect different nation attachment orientations. Items that cluster on component 1 suggest it represents fearful attachment, with items such as “I worry about being abandoned by my country” correlating highly. Items tap into concerns of the attachment figure being absent or rejecting. In analysing the second component it was observed that the items implied a desire for unity with the attachment figure. Most items reflected a secure attachment orientation, but one preoccupied subscale item, “I want to merge completely with my country” also loaded highly on this component; it was deemed that the factor represented secure attachment with preoccupied undertones. The final component had items which converged on independence from the country of origin and self-sufficiency. Items such as “It is very important for me to feel independent from my country” loaded highly onto this factor, which was labelled as dismissive attachment. Reliabilities for the three factors were robust (fearfulness α = .88; secure-preoccupied α = .82; dismissive α = .79). Overall, the hypothesis was supported: the extracted factors reflected attachment orientations of how individuals bonded with their country of origin.

### Hypotheses 2: Correlations with other Independent Variables

Correlations supported the convergent and divergent validity of the derived nation attachment scale. As reported in Table 2, fearful nation attachment was moderately correlated with neuroticism, which aids in validating the former subscale, as both constructs appear to tap into excessive rumination and negative affect. Fearful nation attachment was also positively correlated with general anxious and avoidant attachment models, lending support to the assumption that the subscale measures a conceptually relevant attachment orientation in a separate domain. Fearful nation attachment was also positively correlated with both secure-preoccupied and dismissive nation attachment.

**Table 2:** Correlation Matrix between Variables.

Variable	1	2	3	4	5	6	7	8	9	10
1. Age		−.16*	−.08	−.01	−.17**	−.20**	−.10	−.19**	−.16*	−.01
2. Neuroticism			−.09	−.08	.22**	.51**	−.22**	.47**	.06	.21**
3. Heritage ID				.68**	−.29**	.03	.26**	−.15*	.40**	−.36**
4. Mainstream ID					−.24**	−.03	.20**	−.15*	.33**	−.27**
5. Avoidance						.20**	−.15*	.33**	.00	.13*
6. Anxiety							−.09	.43**	.19**	.08
7. SWB								.03	.27**	−.10
8. Fearful NA									.36**	.34**
9. Secure-Pre.NA										−.25**
10. Dismissive NA										
Mean	30.07	30.28	64.89	64.48	13.96	16.45	16.61	13.72	13.36	16.37
SD	10.33	10.85	12.96	12.83	4.70	4.96	4.58	6.37	4.89	5.47

*Note*:

* *p*<.05,

** *p*<.01.

Secure-preoccupied nation attachment correlated negatively with dismissive nation attachment. Dismissive nation attachment was positively associated with a general avoidant attachment, implying that both measured similar concepts in different domains, and also with neuroticism. Correlations with general attachment models supported a structural coherency to the nation attachment subscales; correlation amongst the subscales implied the possible existence of two underlying higher order factors: model of self, and model of other, as described by Griffin & Bartholomew [7]. The convergent and divergent validities of the nation attachment scale and its three factors were established by the correlations.

### Hypothesis 3: Association of Nation Attachment with Acculturation

Preliminary correlational analysis indicated that nation attachment was associated with differing levels of heritage and mainstream culture identification. Secure-preoccupied nation attachment was associated with increased heritage and mainstream culture identification, whilst dismissive and fearful nation attachment were both negatively correlated with both variables, as shown in Table 2. The hypothesis that nation attachment would account for variance in acculturation over and above the other predictor variables was examined with a hierarchical linear model (HLM), where participants (Level 1) were nested in their country of origin (Level 2). Clustering participants by nationality resulted in thirty-eight Level 2 units. All variables in the model were centred on their grand means. In order to control for confounds on the association of nation attachment with acculturation, we ran a multi-level model with residence status (included as a moderator and coded such that migrants were allocated a value of 1, and participants living in their country of birth a value of −1), neuroticism, general avoidant and anxious attachment as predictors in the first block.

Fearful, secure-preoccupied, and dismissive nation attachment were included in the second block. Interaction terms were created for each nation attachment style and residence status, which were entered in the third block. Results indicated that all three nation attachments predicted heritage culture endorsement over and above the other variables. Secure-preoccupied nation attachment predicted significantly higher levels of heritage culture identification. Conversely, both dismissive and fearful nation attachment predicted lower levels of heritage culture identification. Table 3 illustrates these results.

**Table 3:** Association of Nation Attachment with Heritage Culture Endorsement.

MODEL	*b*	SE *b*
STEP 1		
Neuroticism	−.11	.09
Avoidant attachment	−.83**	.18
Anxious attachment	.36	.19
Country residence status	.04	.85
STEP 2		
Neuroticism	.02	.08
Avoidant attachment	−.60**	.16
Anxious attachment	.25	.17
Country residence status	1.80*	.83
Fearful nation attachment	−.41*	.16
Secure-preoccupied nation attachment	1.24**	.18
Dismissive nation attachment	−.39*	.15
STEP 3		
Neuroticism	.01	.08
Avoidant attachment	−.58**	.16
Anxious attachment	.27	.17
Country residence status	1.61	.86
Fearful nation attachment	−.40*	.17
Secure-preoccupied nation attachment	1.02**	.21
Dismissive nation attachment	−.46**	.16
Fearful nation attachment * Residence status	.18	.15
Secure-Pre. nation attachment * Residence status	−.45*	.21
Dismissive nation attachment * Residence status	−.32*	.16

* *p*<.05.

** *p*≤.005.

**Figure 1:**
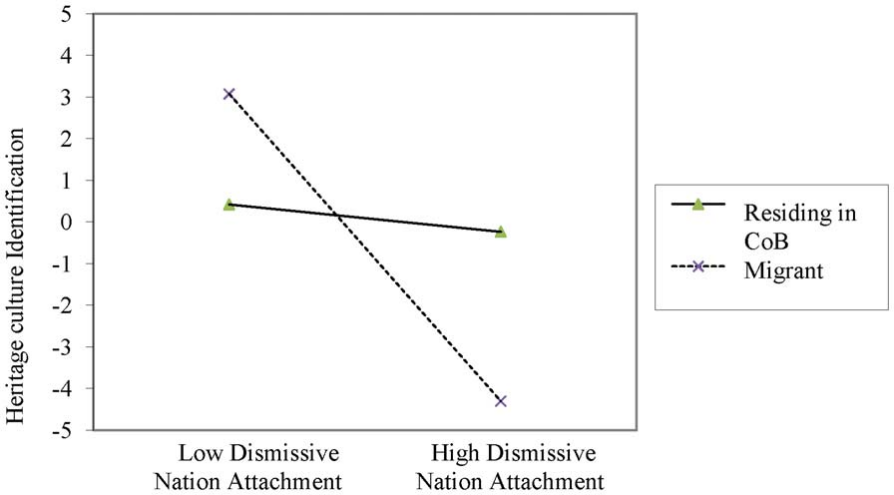
Interaction between resident status and dismissive nation attachment in predicting heritage culture identification levels in Study 1.

**Figure 2:**
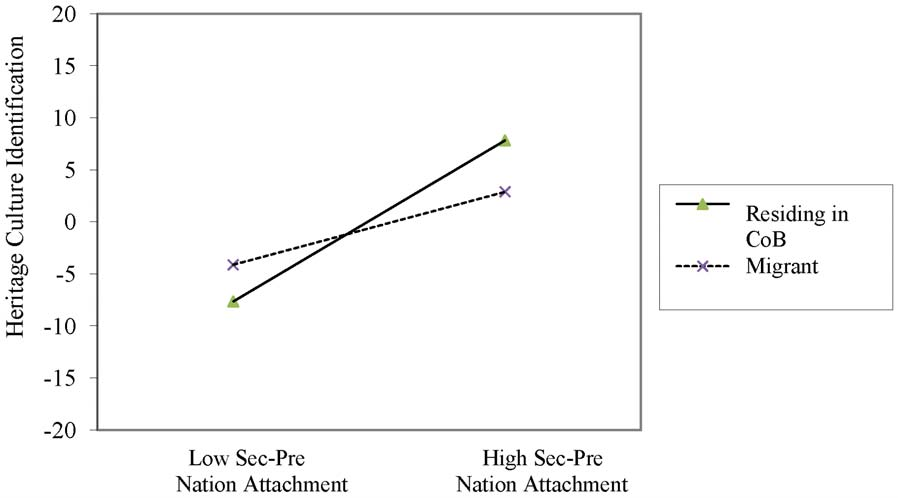
Interaction between resident status and secure-preoccupied nation attachment in predicting heritage culture identification levels in Study 1.

Two interaction terms were significant, country residence status×dismissive nation attachment, and country residence status×secure-preoccupied nation attachment. Simple slope analysis was conducted by re-running the multi-level model on migrants and those residing in their country of birth separately. Dismissive nation attachment was negatively associated with heritage culture identification for migrants, *b* = −.77, *t*(73) = −2.85, *p*<.01, but not for individuals residing in their country of birth (*p* = .41). Conversely, secure-preoccupied nation attachment was significantly positively associated with heritage culture identification for individuals residing in their country of birth, *b* = 1.33, *t*(143) = 7.08, *p*<.001, but not for migrants (*p* = .22). Figures 1 and 2 illustrate the interactions. An identical model with mainstream culture identification as a dependent variable was run for the migrant sample only but did not yield significant results.

### Hypothesis 4: Nation attachment and SWB

SWB was investigated further through hierarchical linear modelling. Similar to the previous model, we controlled for neuroticism, general attachment type, and residence status in the first block. Secure-preoccupied attachment was the only nation attachment type that significantly predicted higher levels of SWB after controlling for confounding variables, as illustrated in Table 4. None of the interaction terms in Step 3 were significant.

**Table 4:** Association of Nation Attachment with SWB.

MODEL	*b*	SE *b*
STEP 1		
Neuroticism	−.09*	.03
Avoidant attachment	−.11	.06
Anxious attachment	−.01	.07
Residence status	−.10	.34
STEP 2		
Neuroticism	−.10**	.03
Avoidant attachment	−.11	.06
Anxious attachment	−.04	.07
Residence status	−.10	.32
Secure-preoccupied nation attachment	.21**	.06
Fearful nation attachment	.08	.07
Dismissive nation attachment	−.01	.06
STEP 3		
Neuroticism	−.10**	.03
Avoidant attachment	−.12	.07
Anxious attachment	−.05	.07
Country residence status	−.11	.34
Fearful nation attachment	.07	.07
Secure-preoccupied nation attachment	.21*	.09
Dismissive nation attachment	−.02	.06
Fearful nation attachment * Residence status	−.03	.06
Secure-Pre. nation attachment * Residence status	.01	.09
Dismissive nation attachment * Residence status	.02	.06

* *p*<.05,

** *p*≤.005.

### Test of Mediation

**Figure 3:**
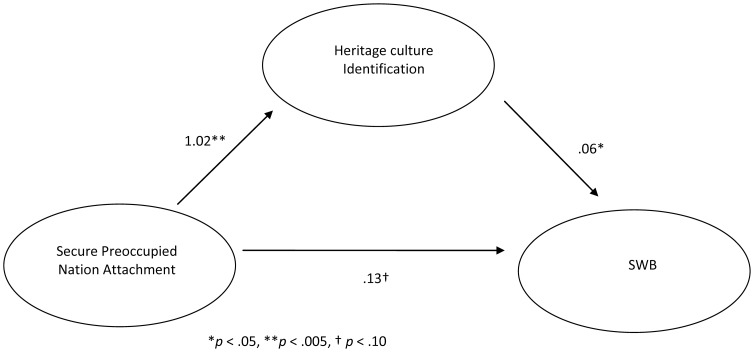
Mediating role of heritage culture identification on the association between secure-preoccupied nation attachment and SWB in Study 1.

After inspecting the associations between secure-preoccupied nation attachment, heritage culture identification, and SWB, the indirect effect of secure-preoccupied nation attachment on SWB through heritage culture identification was tested with a Sobel test. The unstandardized coefficients and standard errors entered into the Sobel test were derived from hierarchical linear models. Results indicated that the association between secure nation attachment and SWB decreased, from *b* = .21 (*t*(159) = 2.87, *p* = .005), to *b* = .12 (*t*(171) = 1.51, *p* = .13), when heritage culture identification was added to the hierarchical linear model; the Sobel test for mediation was significant (*z* = 2.10, *p*<.05), as illustrated in Figure 3.

## Discussion

Results suggested partial support of the five hypotheses. An attachment to nation was elucidated through factor analysis, supporting the first hypothesis. Correlations between general attachment, neuroticism, and nation attachment indicated support for Hypothesis 2 and provided convergent validity for the construct. Nation attachment contributed to a significant portion of variance only in endorsement of a heritage culture identity, thus partially upholding Hypothesis 4 that nation attachment would uniquely predict variations in acculturation. Hypothesis 5, that secure nation attachment styles would be associated with increased levels of SWB, was corroborated. The indirect effect of secure-preoccupied nation attachment through heritage culture identification on SWB was significant. The interaction observed in two samples, with secure-preoccupied nation attachment contributing to a significant portion of variance in the group residing in their country of birth, and dismissive nation attachment contributing to a significant portion of variance for those residing in a mainstream country, implied that the ways in which individuals conceive their attachment to their nation of origin affects identification with their heritage culture. Nation attachment is distinct from the construct of cultural identification as the latter is measured primarily through behavioural items as opposed to the affective and cognitive nature of the nation attachment scale. A second study was conducted on a different participant sample to validate the nation attachment construct.

## Study 2

### Hypotheses

The aims of this study were to replicate and extend the results of Study 1 through (i) testing the nation attachment construct with a different sample, (ii) investigate the construct's concurrent validity with nationalistic attitude, and (iii) investigate its association with flourishing as an extension of SWB. Flourishing has been postulated as a distinct measure of well-being that is separate to life satisfaction, which taps into more evaluative judgements of subjective-well-being [70], [71]; the flourishing construct taps into perceptions of self-efficacy, optimism, success at social relationships, purpose in life, and self esteem. We wanted to investigate whether the link between nation attachment, heritage identification, and SWB also translated to a differing measure of psychological well-being, thus further supporting the results from the first study.

#### Hypothesis 1

Similar to Study 1, we hypothesised that a 3-factor structure of nation attachment would emerge, supporting the validity and significance of a nation attachment framework.

#### Hypothesis 2

Nationalistic attitude will be positively associated with secure-preoccupied nation attachment, and negatively associated with dismissive nation attachment.

#### Hypothesis 3

Nation attachment will account for a significant amount of variance in heritage and mainstream culture endorsement after controlling for nationalistic attitude.

#### Hypothesis 4

Secure-preoccupied nation attachment will be associated with higher levels of SWB and flourishing.

## Method

### Participants

253 participants (female: 125, male: 128) between 18 and 67 years of age (*M* = 28.69, *SD* = 11.41) were used for confirmatory factor analysis (CFA). Due to missing data in other measures, only 210 of the participants were retained for HLM analysis. 18% of participants reported currently living in a country different to that of their birth for between 1 and 34 years (*M* = 8.91, *SD* = 7.53). 82% reported residing in the country of their birth. 22 nationalities were represented, with a large proportion of participants citing residence in South Asian (40%), North American (37%) and European (14%) regions. Distribution of highest attained education was varied, with participants indicating that they had completed or were currently in their first degree (57%), had completed or commenced their postgraduate degree (28%), had completed secondary education (5%), or had completed a professional degree (10%). A high proportion of participants indicated that they were in full-time employment (50%); participants also reported being in full-time education (34%), and unemployed/retired (16%). The majority of participants reported their current relationship status as single (46%), followed by married (34%), along with in a relationship (15%), cohabiting (4%), or divorced/widowed (1%). Due to the small proportion of participants reporting a migrant status, the two groups were collapsed into one, and only heritage culture identification was investigated.

### Materials

#### Nation Attachment

The adapted Nation Attachment questionnaire derived in Study 1 was administered to participants. Participants were asked to indicate the extent to which each of the 30 items was descriptive of themselves on a continuous 5-point Likert scale (1 = *Not at all like me*, 5 = *Extremely like me*). Factor analysis results are included in the Results section.

#### Neuroticism

Participants were asked to indicate the extent of the pertinence of each statement to their personality on the EPQR-S [66] to measure neuroticism (α = .92)

#### Acculturation

The VIA [50] was also included in this study; alphas for the heritage (α = .92) and mainstream (α = .91) subscales were robust.

#### General attachment type

General attachment type was measured using the ECR-S [68] and included as a validation measure for nation attachment. Alphas for both the anxious (α = .65) and avoidant (α = .76) subscales were adequate.

#### Subjective well-being

The SWLS [69] was employed to measure SWB. The reliability of this scale was adequate (α = .88).

#### Flourishing

A measure developed by Diener and colleagues [70] as a counterpart to SWB, the flourishing scale taps into above the baseline human functioning. Participants were asked to indicate the extent of their agreement on a 7-point Likert scale (1 = *Strongly Disagree*, 7 = Strongly Agree) for eight items (α = .92). Sampled items included “I lead a purposeful and meaningful life.”

#### Nationalism

The nationalistic attitude scale was adapted from Kosterman and Feshbach [72]. The nationalistic attitude subscale (α = .89) is composed of eight items and taps into feelings of superiority and dominance of one's country. Participants were asked to indicate on a five-point Likert scale (1 = *Disagree Strongly*, 5 = *Agree Strongly*) the extent of their agreement with each of the statements. For the purposes of this study references to “America” were changed to “my country” (e.g. “Other countries should try to make their government as much like ours as possible”).

### Procedure

The study was conducted online, and the hyperlink to the survey was distributed on a London-based university intranet site, psychology-oriented websites with an international range of participants, at a Midwestern US university, and through MTurk on Amazon (participants were offered $0.25 for completion of the survey). In order to minimise the occurrence of re-sampling, participants were asked not to complete the survey if they had previously participated in the first nation attachment study. IP addresses of participants were inspected to confirm that no duplicates were included.

## Results

### Hypothesis 1: Validating the Nation Attachment construct

To test the validity of the nation attachment construct, confirmatory factor analysis (CFA) was run through AMOS 18. Based on the item loadings derived in Study 1, a model measuring the three latent and inter-correlated variables of fearful, dismissive, and secure-preoccupied nation attachment was specified. Several indices of model fit were inspected, including the chi-square statistic, the chi-square to degrees of freedom ratio, the comparative fit index (CFI), the root mean square error of approximation (RMSEA), and the standardised root mean residual (SRMR). The initial model provided an adequate fit for the data: the chi-square value was significant, (χ^2^(132) = 327.79, *p*<.001), however, it was acknowledged that the chi-square value is susceptible to sample size and thus it is unrealistic to obtain a non-significant value with such a large sample size; χ^2^/*df* = 2.48; CFI = .91; RMSEA = .08 [CI: .07, .09]; SRMR = .07. On the basis of high modification indices and decreased face-validity, three items were removed from the fearfulness subscale (“My country often wants me to be closer than I feel comfortable being,” “I often worry that my country doesn't love me,” and “I worry about having my country not accept me”), and two from the dismissive nation attachment scale (“I am comfortable without a close emotional relationship to my country,” and “I prefer not to depend on my country”).

**Figure 4:**
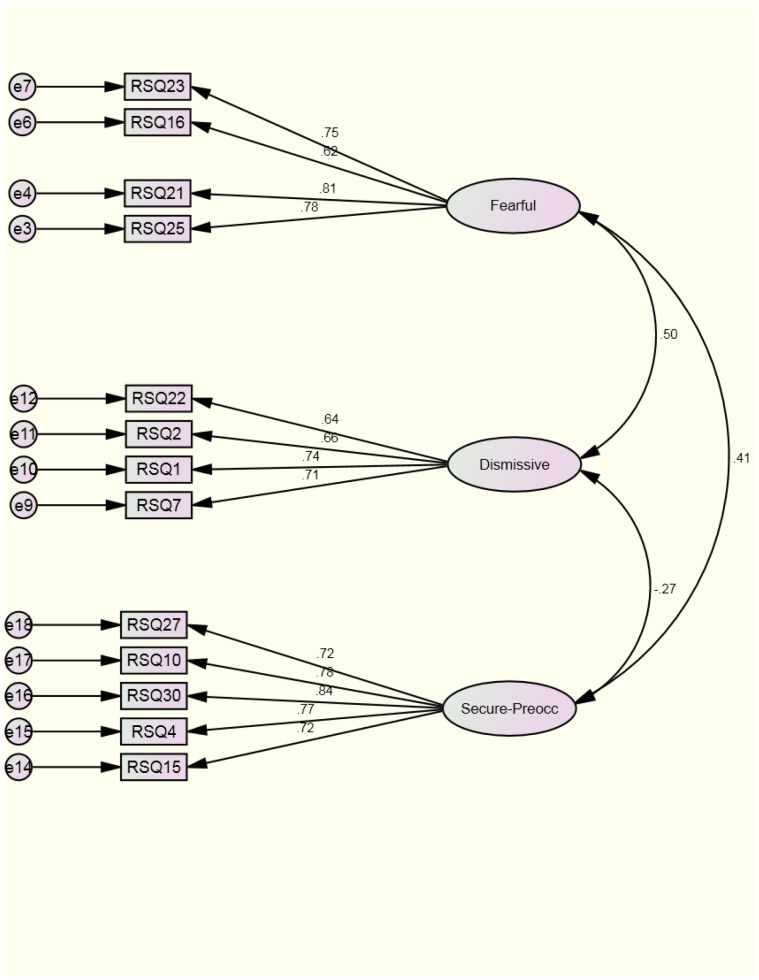
Confirmatory factor analysis model of the latent nation attachment constructs in **Study 2** **.**

Following the removal of five items, the reduced model was assessed as being a better fit according to the guidelines prescribed by Hu and Bentler [73]: the chi-square value was significant (χ^2^(62) = 129.83, *p*<.001); χ^2^/*df* = 2.10; CFI = .95; RMSEA = .07 [CI: .05, .08]; SRMR = .06. Model fit was significantly improved with the removal of the items, χ^2^Δ (70) = 197.80, *p*<.001. Figure 4 illustrates the final nation attachment model, with four items tapping into dismissive and fearful nation attachment, and five into the secure-preoccupied construct. Item factor loadings were greater than .62 and significant (see Table 5 for retained items) and Cronbach's alphas remained robust after the removal of items in the fearfulness (α = .83) and dismissive scales (α = .78); the reliability of the secure-preoccupied subscale was satisfactory in the second sample (α = .88). The model therefore demonstrated the validity of the nation attachment construct in a second sample; subsequent analyses were based on the reduced nation attachment scale.

**Table 5:** Retained Items for the Nation Attachment Scale (Study 2).

Item	Factor Loadings
*Fearful Nation Attachment*	
I often worry that my country won't want to stay with me	.81
I worry about being abandoned by my country.	.75
I find that my country is reluctant to get as close as I would like.	.77
I worry that my country doesn't value me as much as I value my country.	.62
*Secure-Preoccupied Nation Attachment*	
I know my country will be there when I need it to be.	.74
I want to merge completely with my country.	.76
I am comfortable depending on my country.	.78
I find it relatively easy to get close to my country.	.84
I am comfortable having my country depend on me.	.72
*Dismissive Nation Attachment*	
It is very important for me to feel independent from my country	.67
I prefer not to have my country depend on me.	.63
I find it difficult to depend on my country of origin.	.74
I am not sure I can depend on my country to be there when I need it to be.	.73

### Hypotheses 2 and 3: validity and heritage culture identification

Study 2 sought to extend the convergent and discriminant validity of the nation attachment construct. As illustrated in Table 6, secure-preoccupied and fearful nation attachments were positively correlated with nationalistic attitude. Dismissive nation attachment was negatively associated with nationalistic attitude. The pattern of intercorrelation between the three nation attachment styles was replicated from the first study.

**Table 6:** Correlation Matrix between Variables in Study 2.

Variable	1	2	3	4	5	6	7	8	9	10	11
1.Age		−.14*	.15*	−.19**	−.12	−.02	.12	.09	−.02	.16*	−.07
2.Neuroticism			−.12	.22**	.40**	−.34**	−.48**	−.11	.40**	−.03	.29**
3.Heritage ID				−.36**	−.10	.33**	.47**	.32**	−.08	.33**	−.23**
4.Avoidance					.25**	−.22**	−.37**	−.01	.30**	.00	.07
5.Anxiety						.00	−.19**	.21**	.46**	.25**	.19**
6.SWB							.65**	.32**	.02	.32**	−.18**
7.Flourishing								31**	−.17**	.26**	−.24**
8.Nationalistic Att.									.27**	.66**	−.15*
9.Fearful NA										.35**	.41**
10.Secure-Pre. NA											−.23**
11.Dismissive NA											
Mean	28.69	28.86	65.85	14.17	16.33	17.66	45.06	24.01	8.31	14.74	10.24
SD	11.41	10.83	16.26	5.03	4.65	5.14	9.23	8.411	4.02	5.62	4.15

*Note*:

* *p*<.05,

** *p*<.01.

We reconstructed the hierarchical linear model from Study 1 to investigate the association of nation attachment with heritage culture identification when controlling for confounding variables, with individuals nested in their reported nationality groups. As the proportion of migrants was lower in this study, residence status and the interaction between residence status and nation attachment were not examined. General attachment style, neuroticism, and nationalistic attitude were entered as control variables in the first block. Fearful, secure-preoccupied, and dismissive nation attachment were entered in the second block. In this model, secure-preoccupied nation attachment predicted significantly higher levels of heritage culture identification, over and above other variables, including nationalistic attitude; Table 7 illustrates these results.

**Table 7:** Association of Nation Attachment with Heritage Culture Endorsement (Study 2).

MODEL	*b*	SE *b*
STEP 1		
Neuroticism	−.01	.11
Avoidant attachment	−1.02**	.20
Anxious attachment	−.32	.25
Nationalistic attitude	.67**	.12
STEP 2		
Neuroticism	.06	.11
Avoidant attachment	−.98**	.20
Anxious attachment	−.27	.25
Nationalistic attitude	.41*	.16
Fearful nation attachment	−.38	.34
Secure-preoccupied nation attachment	.66**	.25
Dismissive nation attachment	−.29	.28

* *p*<.05,

** *p*≤.01.

### Hypothesis 4: Nation attachment, SWB, and Flourishing

The relationship between nation attachment and SWB was further investigated in the second sample with a replicated HLM from Study 1 that controlled for neuroticism, general attachment type, culture, and nationalistic attitude in the first block, and nation attachment in the second. The results were similar to the previous study, with secure-preoccupied being the only nation attachment type that was significantly associated with higher levels of SWB, as illustrated in Table 8.

**Table 8:** Association of Nation Attachment with SWB (Study 2).

MODEL	*b*	SE *b*
STEP 1		
Neuroticism	−.14**	.03
Avoidant attachment	−.16*	.07
Anxious attachment	.08	.08
Nationalistic attitude	.17**	.04
STEP 2		
Neuroticism	−.30**	.04
Avoidant attachment	−.20**	.07
Anxious attachment	.07	.08
Nationalistic attitude	.08	.05
Secure-preoccupied nation attachment	.18*	.08
Fearful nation attachment	.06	.11
Dismissive nation attachment	−.07	.09

* *p*<.05,

** *p*<.005.

An identical HLM was constructed to assess the association of nation attachment and flourishing, as depicted in Table 9. Results indicated that secure-preoccupied nation attachment was significantly positively associated with flourishing after controlling for confounding variables.

**Table 9:** Association of Nation Attachment with Flourishing (Study 2).

MODEL	*b*	SE *b*
STEP 1		
Neuroticism	−.29**	.06
Avoidant attachment	−.49**	.11
Anxious attachment	−.12	.13
Nationalistic attitude	.31**	.06
STEP 2		
Neuroticism	−.29**	.06
Avoidant attachment	−.44**	.11
Anxious attachment	−.13	.13
Nationalistic attitude	.23*	.09
Secure-preoccupied nation attachment	.36**	.18
Fearful nation attachment	−.07	.13
Dismissive nation attachment	−.01	.15

* *p*<.05,

** *p*≤.01.

### Tests of Mediation

**Figure 5:**
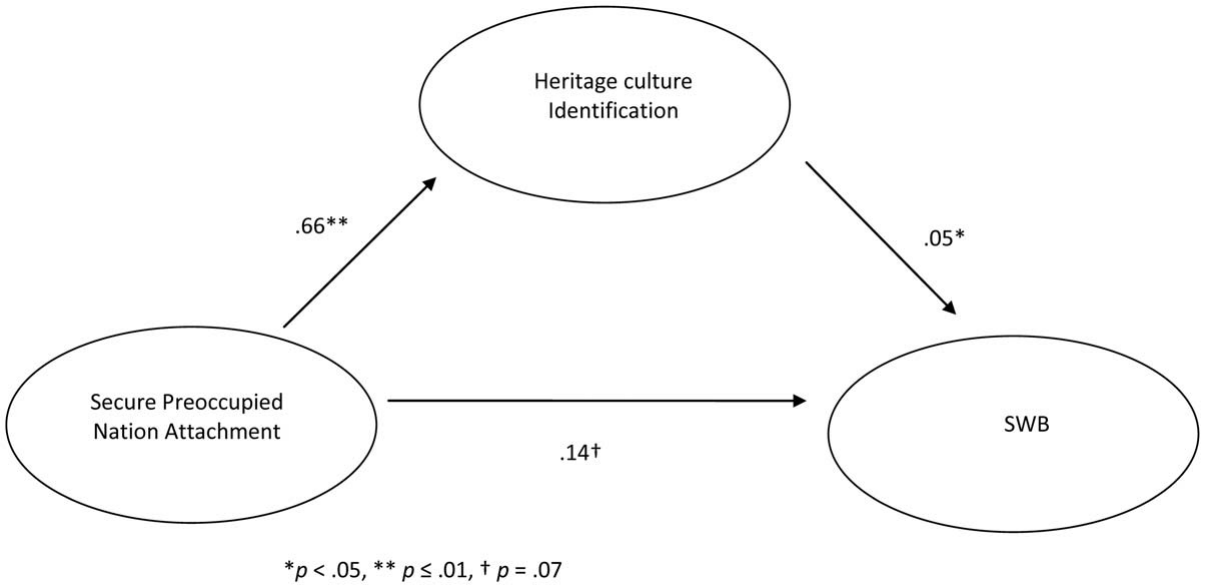
Mediating role of heritage culture identification on the association between secure-preoccupied nation attachment and SWB in **Study 2** **.**

**Figure 6:**
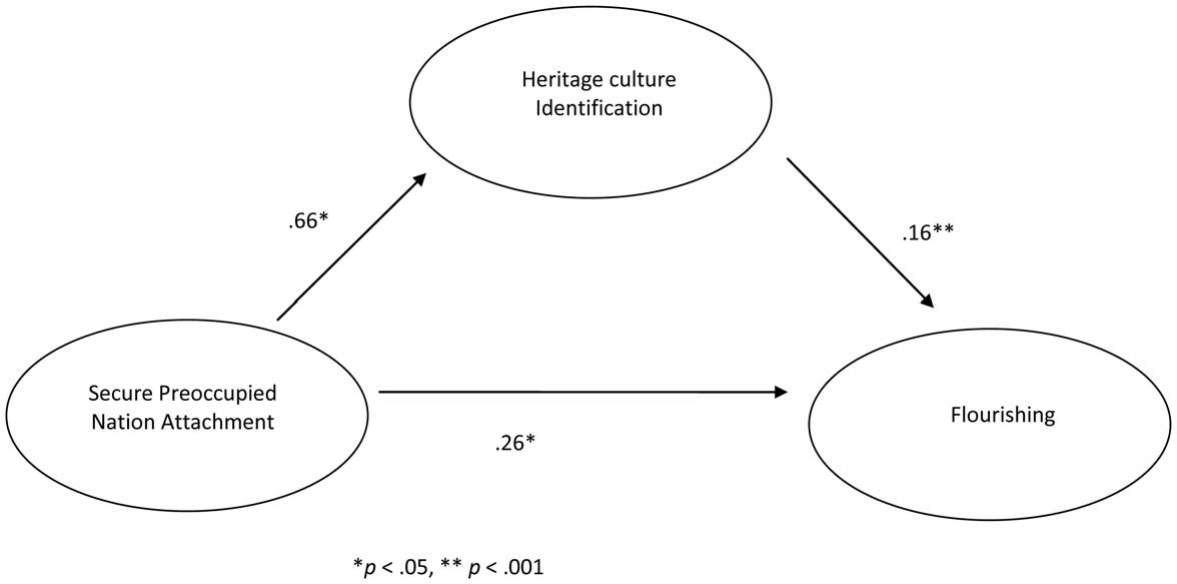
Mediating role of heritage culture identification on the association between secure preoccupied nation attachment and flourishing in **Study 2** **.**

In order to assess the replicability of results from Study 1, the indirect effect of secure-preoccupied nation attachment on SWB through heritage identification was tested with a Sobel test; all values entered to the test were derived from hierarchical linear models. Including heritage culture identification in the model decreased the association between secure-preoccupied nation attachment and SWB, from *b* = .18 (*t*(195) = 2.24, *p*<.05), to *b* = .14 (*t*(194) = 1.81, *p* = .07). The Sobel test approached significance, *z* = 1.82, *p* = .07. Figure 5 illustrates the mediation effect of heritage culture identification on the association between secure-preoccupied nation attachment and SWB. In order to extend these results, a mediation model was also tested on the association between secure-preoccupied nation attachment and flourishing, with heritage culture identification as a mediator; inclusion of this variable resulted in a decrease in the association between secure-preoccupied nation attachment and flourishing, from *b* = .36 (*t*(192) = 2.74, *p* = .01), to *b* = .26 (*t*(186) = 2.09, *p* = .04). The Sobel test was significant, *z* = 2.20, *p*<.05, and this mediation pathway is illustrated in Figure 6.

## Discussion

The second study provided support for the nation attachment construct through CFA. The resulting reduced model, which improved on the scales derived in Study 1, provided an adequate fit for the data. The reliabilities of the scales remained robust after excluding items. Hypothesis 2 was supported with the correlational patterns between nation attachment constructs and nationalistic attitudes. Hierarchical linear models testing the association of nation attachment with heritage culture identification after including the control variables was partially replicated in this study; the lack of an association between dismissive nation attachment and heritage identification is attributed to a lower proportion of migrant participants. Secure-preoccupied nation attachment was significantly associated with flourishing through heritage culture identification, and this mediation pathway approached significance for secure-preoccupied nation attachment and SWB.

## General Discussion

### Attachment to Nation of Origin Scale

A model of attachment to nation with three conceptually coherent factors was obtained from the adapted RSQ. Becker and colleagues [74] reported a three-factor model which included the present study's fearful and secure-preoccupied factors. Further support comes from Bäckström and Holmes [10], who reported in a factor analysis of the RSQ that a three-factor model provided the best fit for their data. The three distinct factors reflected a secure-preoccupied attachment to one's nation, which converged on a desire to establish emotional and dependent links and to merge with one's nation, a fearful attachment that tapped into ruminations about the stability of the attachment bond between self and nation, and a dismissive attachment which can be interpreted as either a marked desire to separate oneself from one's nation of origin and establish independence and self-sufficiency, or as the lack of an attachment bond. This model was further validated in an independent sample using CFA. The resulting model was adequate [73].

Inter-correlation between the nation attachment orientations was consistent with other research [10]. It can also imply the presence of higher order factors: the self and other models. In this light, secure-preoccupied nation attachment represented positive self and other models, with an undertone of a negative self model, which accounted for its association with fearful nation attachment. The presence of both secure and preoccupied elements in this nation attachment type is intriguing, as the preoccupied category in the general attachment literature has a negative component [6], [8]. This was replicated in the current research, as illustrated with the item of one's desire to merge with their country of origin, despite the construct's positive association with measures of psychological adjustment in both studies. This is further expounded upon in the section on nationalistic attitudes. Fearful nation attachment was also associated with dismissive nation attachment, which is characterised by positive self and negative other representations. The negative association between secure-preoccupied and dismissive nation attachment lends further credence to the existence of discrete attachment orientations which circumscribe the relationships that individuals form with their nation of origin.

### Nation Attachment and General Attachment Models

Taking example from attachment models in a similar field of symbolic religious attachment [75], [76], the hypothesis that nation attachment orientations would be associated with their respective general attachment models was supported. Dismissive nation attachment was found to correlate with general avoidant attachment models, implying that individuals who reported avoidant attachment were also likely to report a dismissive attachment to their nation. Fearful nation attachment correlated with both anxious and avoidant attachment models, reflected in this particular orientation representing both the ruminating tendencies of anxious attachment, and the characteristic negative other model of avoidant attachment. Secure-preoccupied nation attachment correlated positively with general anxious attachment, underscoring the preoccupied element.

### Nation Attachment and Nationalistic Attitudes

Correlations between the nation attachment construct and nationalistic attitudes measures reinforce its convergent validity. Secure-preoccupied nation attachment was positively correlated with nationalistic attitude; it is important to note that secure-preoccupied nation attachment contributed to variance in heritage culture identification over and above nationalistic attitude endorsement, thereby providing support that it taps into separate and distinct components of the relationship that individuals conceptualise towards their country. This finding serves to distinguish nation attachment, particularly secure-preoccupied nation attachment, as a more positive aspect of ingroup attachment than nationalism, which is defined by beliefs of superiority. The items that measure the nation attachment construct focus on emotional attachment to the symbolic manifestation of the ingroup that is independent of outward prejudice [77]. The preoccupied item that expressed a desire to merge with one's country on the secure-preoccupied scale implies a heightened dependency on one's symbolic representation of their country of origin, which could translate into a more collective or interpersonal sense of self [78] that incorporates within it this representation. Future research could seek to test individuals' self-concepts when taking into account one's country of origin. Dismissive nation attachment correlated negatively with nationalistic attitudes, as hypothesised: a lack of attachment to one's country should also be associated with decreased interest and conviction in the country's culture and systems. Fearful nation attachment was also associated with increased nationalistic attitude. This result was unexpected, but taken together with the finding of the association of nationalistic attitudes with external threats such as loss of territory [79], provides insight into the uncertainty of availability of the other that characterises fearful nation attachment. The inclusion of nationalistic attitude in Study 2 provided further support for the validity of the nation attachment construct.

### Nation Attachment and Heritage Culture Identification

Initial investigation of the correlations of heritage and mainstream culture identification with the three nation attachment orientations yielded results that were interpreted as the former being associated with nation attachment along two distinct trends: individuals who reported secure-preoccupied attachment were likely to identify with both their heritage and host countries, implying an integrated acculturation strategy, whilst those who reported dismissive or fearful attachment were negatively associated with both cultures, implying a marginalised strategy. Anomalously, neither dismissive nor fearful nation attachment were significantly associated with SWB, contravening the established pattern in the wider literature that marginalised experiences are associated with lower levels of SWB [48], [53], [80]. The lack significant findings for migrant participants could be attributed to the sample size of the group.

Hierarchical linear models indicated two differing attachment styles that individuals endorsed towards their nation of origin, dismissive and secure-preoccupied. In Study 1, the interaction of dismissive nation attachment with resident status was significant in the association with heritage culture identification. The lack of significant results for dismissive nation attachment in Study 2 could be attributed to a lower proportion of participants reporting residing in a country different to that of their birth. For participants who reported residing in a mainstream culture in Study 1, dismissive nation attachment contributed significantly over and above the influence of the control variables. A dismissive nation attachment bond predicted lower levels of beliefs, values, and behaviours associated with one's heritage culture. In terms of acculturation, for the migrant sample, lower endorsement of heritage culture translates into two possible orientations, assimilation or marginalisation. As mainstream culture identification was not found to be significant in the current regression model, speculations as to which acculturation orientation is predicted by dismissive nation attachment remain. A tentative conjecture can be made when correlational relationships are taken into account, as a dismissive nation attachment was found to be correlated with lower identification of both cultures, implying a marginalised strategy.

Secure-preoccupied nation attachment also played a significant role in predicting heritage culture identification for participants who reported residing in their country of birth in both studies. These results imply that individuals who formed such an attachment bond with their nation of origin were more likely to maintain their culture's beliefs, values, and traditions. Secure-preoccupied nation attachment, and its focus on an emotional and dependent bond between individual and nation, was associated with higher levels of identification with one's heritage culture. The causal pathway, whether secure-preoccupied attachment is an important feature of individuals who choose to remain in their country of birth, or whether the attachment is formed subsequent to prolonged residence in one's community, and thus nation of origin, is an intriguing one, which is yet to be investigated. Preliminary results do confirm that differing nation attachments offer one level of insight into how individuals approach their heritage culture identity. Considered collectively, the present findings contribute to the growing research on how attachment styles are associated with acculturation trends [61], [62], indicating that the ways that individuals identify with their heritage culture is, in part, associated with their relationship to their nation of origin.

### Nation Attachment and Psychological Well-being

The positive association between secure-preoccupied nation attachment, SWB, and flourishing, was noteworthy. The HLMs indicated that secure-preoccupied nation attachment contributed to variance in these two measures of psychological well-being over and above the confounding variables. A mediational model was supported in Study 1 between secure-preoccupied nation attachment, heritage identification, and SWB; this model approached significance in Study 2. The implication of this association with psychological adjustment deserves attention; maintaining and cultivating a close attachment relationship with one's nation of origin is associated with heritage identification, which in turn, correlated with psychological adjustment, when interpreted in terms of global satisfaction with one's life and flourishing in society. The nation attachment construct also provides another facet to the link between acculturation, particularly maintenance of the heritage culture, and psychological well-being [81].

### Limitations and Further Research

There were several limitations of the present studies were numerous, but they did not detract from the overall implications. The exact structure of the nation attachment construct might be better elucidated from the design of a specific nation attachment scale, rather than the use of an adapted scale, which has been found to optimally fit data into three-factor models [10]. Although the RSQ was developed as a flexible measure which captures several differing models of attachment, this instrument was previously employed to assess human interpersonal relationships, and may not shed light onto all aspects of the nation attachment construct. Furthermore, it was originally developed to measure a four-category model of attachment; despite its plasticity, the existing categories do impact the resulting perspective on nation attachment, simply by the design of the items tapping into preoccupied, secure, fearful, or dismissive attachment types. Also, the target attachment figure in all the original items is an active agent rather than a symbolic construct. However, this model has been applied to human-pet relationships [82], which provides further support for its validity as measuring differing attachment targets, along with consistent results for nation attachment types in Studies 1 and 2. Our results implied that some of the items translated across were unwieldy (indeed, items from the Nation Attachment scale were eliminated in both Studies 1 and 2 based on their low face-validity). Future research on nation attachment could avoid the limitations imposed specifically by the RSQ through two separate research pathways: first, through adapting different attachment measures, such as the ECR-S [68] to explore the facets of this construct, and, second, through constructing a nation attachment measure, drawing on attachment theory, and piloting it on participants using both quantitative and qualitative measures. Through developing a novel nation attachment measure future research should also focus on the formation of nation attachment models, both in migrants and non-migrants, as this was beyond the scope of the present research which focused only on existing models.

Validation of the proposed construct of nation attachment obtained with the present adapted scale should also be conducted. The merits of the present research would be improved if the test-retest reliability, divergent, and convergent validity of the adapted RSQ scale were further confirmed. The latter have been confirmed to an extent in the present studies with the inclusion of general attachment models, neuroticism, and nationalistic attitudes, but can further be validated with the use of numerous measures. Convergent validity assessment can be conducted through several methods, including items that explicitly tap into how individuals conceptualise their heritage nation and their bond to it, as well as gathering more data from larger samples, and cross-cultural analysis. The present research took into account variations across differing cultures by adopting a multilevel approach wherein individuals were nested in their nationality groups, however, as the number of individuals per Level 2 units was small, it would be of particular interest to investigate with a larger participant sample whether nation attachment distribution differs depending on culture, as findings from Schmitt and colleagues [83] imply that the distribution of general attachment orientations are significantly skewed towards preoccupied attachment in East Asian cultures. The present data can also pave the way for further research in an experimental paradigm. Primes of nations as attachment figures, including imagery that personify nations as symbolic caregivers, can be employed to investigate several avenues, including the effect on the acculturation orientations of both heritage and mainstream samples, endorsement of multicultural ideology, and patriotic and nationalistic attitudes.

Finally, the term ‘nation’ has been problematic, and has received much attention [84]. No distinction was made between civic or ethnic conceptualisations of nation during data collection or analysis [85], as we extrapolated the meaning that participants drew from ‘country of origin’ in the nation attachment scale by tying it to their nationality. In this way the aim was to tap into the attachment that individuals form with their country of origin without activating nationalistic attitudes that using a term such as “my nation” would have. Future research should focus on defining what individuals interpret when asked about their nation as opposed to their country attachment.

### Conclusions

The metaphor of a nation functioning as a super family subsists as a socially constituted image in several languages. The present studies sought to empirically test the presence of such a symbolic relationship in the attachment models that individuals hold. Results implied that individuals conceptualised their attachment to their nation of origin in three distinct styles. Fearful, secure, and dismissive nation attachment predicted heritage culture identification in the two groups; a mediational link was found between secure-preoccupied nation attachment through heritage identification to SWB and flourishing. Research can further investigate the effects of this relationship, and incorporate it into the attachment framework, capturing the elusive and often bewildering bonds that individuals form with their nation, echoed in the contemplations of poet Mikhail Lermontov, “I love my fatherland, but with a peculiar love/my better judgement cannot conquer it.”
